# Crystal structure of poly[(μ_3_-thio­cyanato-κ^3^ *N*:*S*:*S*)(tri­methyl­phosphine sulfide-κ*S*)copper(I)]

**DOI:** 10.1107/S1600536814021412

**Published:** 2014-10-04

**Authors:** Peter W. R. Corfield

**Affiliations:** a Department of Chemistry, Fordham University, 441 East Fordham Road, Bronx, NY 10458, USA

**Keywords:** crystal structure, phosphine sulfide, thio­cyanate, coordination polymer, copper(I) complex

## Abstract

In the title compound, [Cu(NCS)(C_3_H_9_PS)]_*n*_, the thio­cyanate ions bind the Cu^I^ atoms covalently, forming infinite –Cu—SCN—Cu– chains parallel to the *a* axis. Each Cu^I^ atom is also coordinated to a tri­methyl­phosphine sulfide group *via* a Cu—S bond. Two crystallographically independent chains propagate in opposite directions, and are held together in a ribbon arrangement by long bonds between Cu^I^ atoms in the first chain and thio­cyanate S atoms in the second, with Cu—S = 2.621 (1) Å. The geometry around the Cu^I^ atoms in the first chain is distorted tetra­hedral, with angles involving the long Cu—S bond much less than ideal, and the S—Cu—N angle between the phosphine sulfide S atom and the thio­cyanate N atom opening out to 133.19 (9)°. Each Cu^I^ atom in the second chain appears to be disordered between two positions 0.524 (4) Å apart, with occupancy factors of 0.647 (6) and 0.353 (6). The Cu^I^ atom in the major site is in a distorted trigonal–planar configuration, with the S—Cu—N angle between the phosphine sulfide and the thio­cyanate N atom again opened out, to 137.01 (15)°. The Cu^I^ atom in the minor site, however, forms in addition a long bond [Cu—S = 2.702 (5) Å] to the phosphine sulfide of the first chain, not the thio­cyanate S atom, to provide a further link between the chains.

## Abstract 2

The thio­cyanate ions bind the CuII atoms covalently, forming infinite –Cu—SCN—Cu– chains parallel to the *a* axis. Two crystallographically independent chains propagate in opposite directions, and are held together in a ribbon arrangement by long bonds between Cu^II^ atoms in the first chain and thio­cyanate S atoms in the second.

## Chemical context   

The synthesis and metal coordination reactions of phosphine sulfides is of continuing inter­est (Sues *et al.*, 2014 ▶; Tiedemann *et al.*, 2014 ▶). The title compound was synthesized by Tiethof *et al.* (1974 ▶) as part of an early series of studies on the coord­in­ation chemistry of copper(I) with these sulfur ligands, which established the importance of trigonal–planar coordination for copper(I), then still rare. Indeed, the structure of the cation in [Cu(Me_3_PS)_3_]ClO_4_ (Eller & Corfield, 1971 ▶) was the first example of trigonal–planar coordination in a monomeric copper complex.
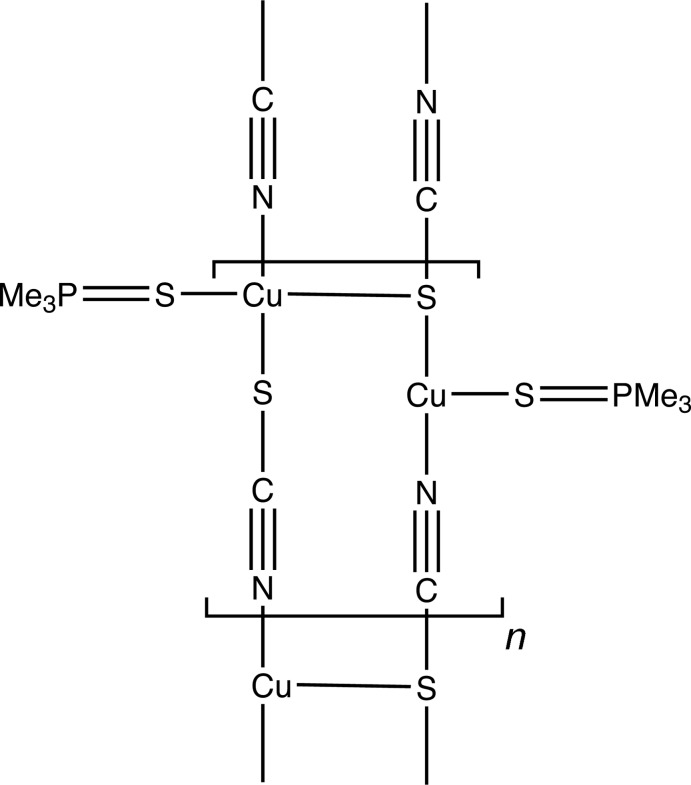


Use of the pseudohalide thio­cyanate in the synthesis of coordination compounds is a well-used route in the design of polymeric structures. Early papers on copper thio­cyanate polymers involving amine adducts include Raston *et al.* (1979 ▶) and Healy *et al.* (1984 ▶). More recent studies include papers on the optical properties of self-assembled amine copper(I) thio­cyanate complexes by Niu *et al.* (2008 ▶) and Miller *et al.* (2011 ▶), as well as studies on magnetic properties of a number of similar copper(II) complexes by Machura *et al.* (2013 ▶).

## Structural commentary   

The previously determined structure of [Cu(Me_3_PS)Cl]_3_ (Tiethof *et al.*, 1973 ▶) consists of a six-membered ring of alternating Cu and S atoms, with trigonal–planar coordination for the Cu^I^ atoms completed by bonds to a Cl atom. It was noteworthy that the Me_3_PS phosphine sulfide ligands bridged the Cu^I^ atoms to form the ring, and not the chlorine atoms as might have been expected. The structure of [Cu(Me_3_PS)SCN] was undertaken to determine whether this trimeric structure persisted in the presence of the thio­cyanate ligand.

The present work determined that tri­methyl­phosphine­copper(I) thio­cyanate crystallizes as a one-dimensional polymer, rather than as the discrete trimers found for the chloride analog. Thio­cyanate ions bind to two separate copper(I) atoms through Cu—N and Cu—S bonds. In the crystal, the two Cu^I^ atoms are related by translation, which leads to the formation of infinite —Cu—SCN—Cu— chains parallel to the *a* axis. Although the Cu—N—C angles are approximately linear, the Cu—S—C angles are bent considerably, as expected (see Table 1 ▶). Each Cu^I^ atom is also coordinated to a terminal Me_3_PS group *via* a Cu—S bond.

Two crystallographically independent chains propagate in opposite directions, and are held together in a ribbon arrangement by long Cu—S bonds between the chains. While no disorder was seen in the first chain, each Cu^I^ atom in the second chain appears to be disordered over two positions, Cu2*A* and Cu2*B*, 0.524 (4) Å apart, with occupancy factors of 64.7 (6)% and 35.3 (6)%, and slightly different coordination spheres (Fig. 3).

**Figure 1:**
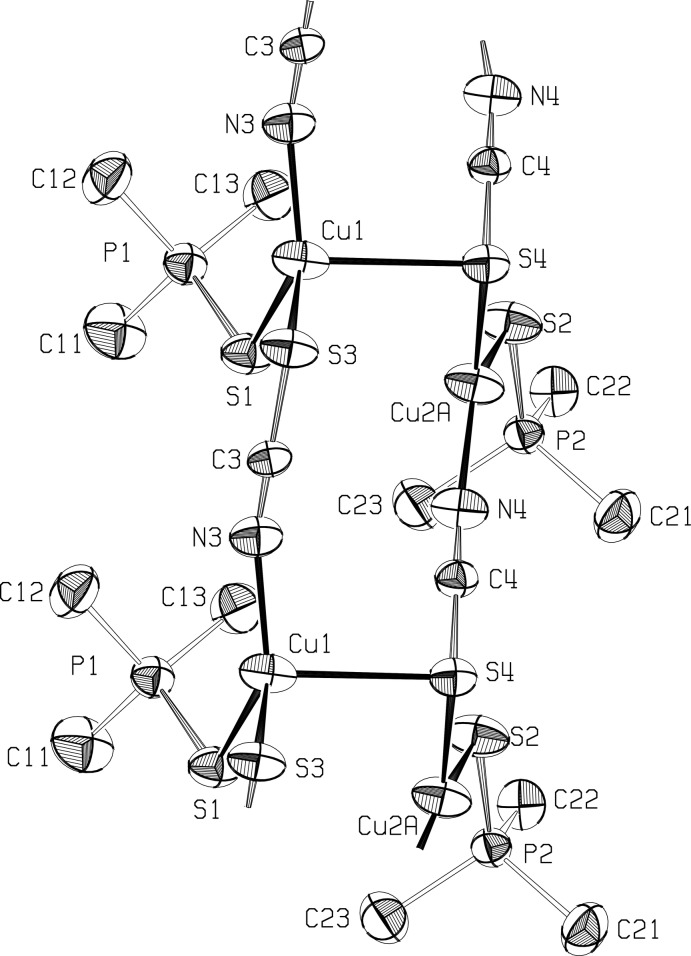
The ribbon structure of the title polymer, with displacement ellipsoids drawn at the 50% level, showing Cu2 in position *A*. Hydrogen atoms are omitted.

Cu^I^ atoms in the first chain bind to thio­cyanate sulfur atoms in the second, with Cu1—S4 = 2.621 (1) Å. Also, one of the disordered Cu^I^ atoms in the second chain forms a long bond to the phosphine sulfide of the first chain, with Cu2*B*—S1 = 2.702 (5) Å, forming another link between the chains (Figs. 1 ▶ and 2 ▶), and a ladder arrangement that is seen also in one of the structures in Healy *et al.* (1984 ▶) and in Niu *et al.* (2008 ▶). The Cu—Cu distances across the chain are 3.656 (3) Å for Cu1—Cu2*A* and 3.351 (5) Å for Cu2*B*. ▶

In the current structure, the two independent Me_3_PS groups are non-equivalent: the group in the second chain, C21–C23, P2 and S2, is terminal, while that in the first chain, C11–C13, P1 and S1, forms an asymmetric bridge between Cu1 and the minor component atom Cu2*B*. This may explain the observation of two different P=S stretching bands in the infra-red spectrum, see below. The two thio­cyanate groups are also non-equivalent, with both S3 and S4 bonded to Cu and N atoms, but S4 forming an additional long bond to Cu1. The non-equivalent groups do not show significant differences in geometry, however (Table 1 ▶).

The geometry around Cu1 atoms, in the first chain, is distorted tetra­hedral, with angles involving the long Cu1—S4 bond much less than ideal, and the S1—Cu1—N3 angle between the phosphine sulfide and the thio­cyanate N atom increased to 133.19 (9)°. The geometry around the disordered Cu^I^ atom in the major site, Cu2*A*, is in a distorted trigonal–planar configuration, with the S2—Cu2*A*—N4 angle between the phosphine S and the thio­cyanate N atoms again opened out, to 137.01 (15)°. Atom Cu2*B* has an irregular tetra­hedral configuration. The geometry at the three-coordinated sulfur atoms S1 and S4 is trigonal–pyramidal rather than trigonal–planar, with the sum of the angles at S1 = 303.2°, while at S4 the sum is 294.8° for angles involving Cu2*A* and 281.0° for angles with Cu2*B*.

## Supra­molecular features   

A packing diagram viewed down the a^*^ axis is shown in Fig. 4 ▶. There are no strong inter­actions between the chains, and all inter­molecular contacts appear normal. The shortest inter­molecular contacts are H13*A*⋯H23*B*(1 + *x*, *y*, *z*), at 2.53 Å, and H12*A*⋯H12*A*(1 − *x*, 1 − *y*, 1 − *z*) at 2.57 Å. All other H⋯H contacts are greater than 2.7 Å.

## Database survey   

Entry CMPSCU in the Cambridge Structure Database (CSD) is taken from the abstract of our presentation at the 1973 Winter Meeting of the American Crystallographic Association. No coordinates were given.

A search of the database with the fragment Cu—S—C N—Cu fingered 100 analyzable structures with 164 thio­cyanate groups. The average thio­cyanate geometries were: C N = 1.152 (17), S—C = 1.65 (2)Å; S—C N = 178.2 (14)°. Corresponding parameters in the present structure are indistinguishable from these average values. A much greater spread is seen in average parameters involving Cu, reflecting the diversity of chemical inter­actions in these structures. For example, average values for Cu—S distances are 2.5 (2) Å, with a range from 2.20 to 3.12 Å.

## Synthesis and crystallization   

Details of the synthesis and characterization of the title compound are given in Tiethof *et al.* (1974 ▶), which describes the preparation and characterization of a series of copper(I) complexes with tertiary phosphine sulfide, phosphine selenide, and arsine sulfide ligands. Solid LiSCN (0.59 mmol) was stirred with 7 mL of a solution of 0.63 mmol of [Cu(Me_3_PS)_3_]BF_4_ in aceto­nitrile for 30 min. The resultant solid was collected, washed with ether, dried *in vacuo*, and characterized by C, H, and N elemental analysis. The infra-red spectrum of a solid sample in a Nujol mull gave bands attributed to P=S stretching at 543 and 546 cm^−1^. These frequencies are similar to the frequency of 540 cm^−1^ observed for [Cu(Me_3_PS)_3_]BF_4_, where the phosphine ligands are terminally bonded to copper as in the present structure, and significantly different from the P=S frequency of 564 cm^−1^ observed for the free ligand, Me_3_PS.

## Refinement details   

**Figure 3:**
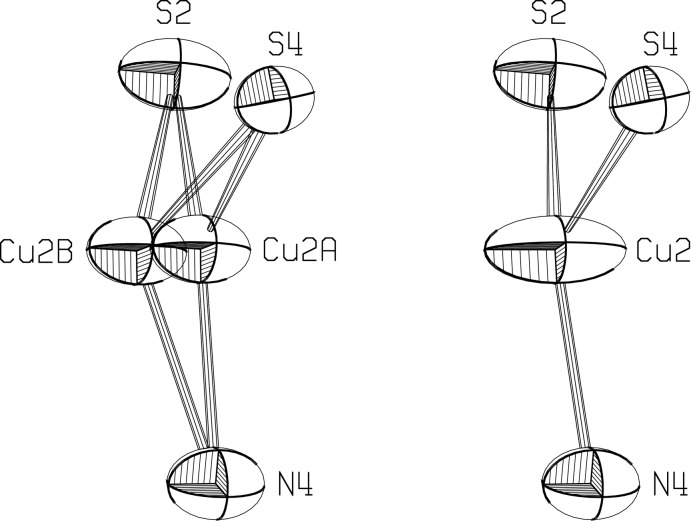
The Cu2 ellipsoids after and before the disordered model was introduced. Displacement ellipsoids are drawn at the 50% level.

**Figure 4:**
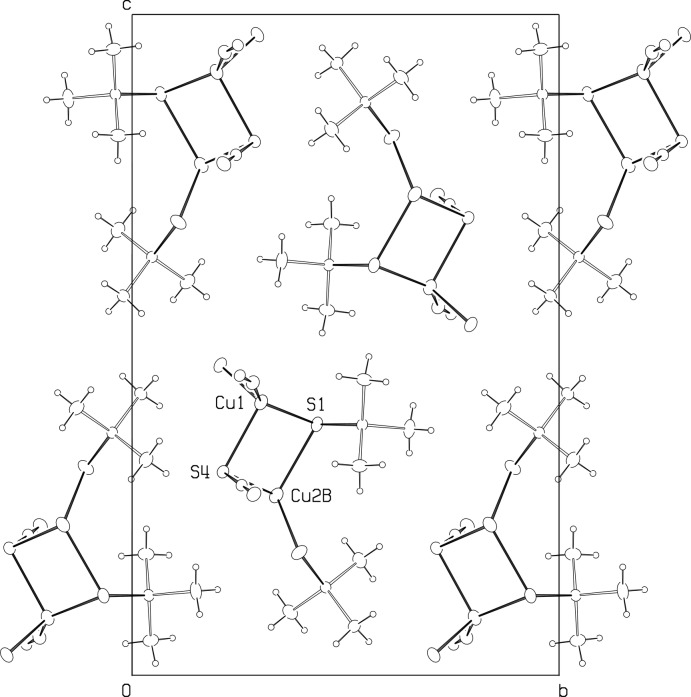
Packing of the title complex, viewed along the a^*^ axis, with ellipsoid outlines at 30% probability.

Initial refinements with anisotropic displacement parameters for all non-hydrogen atoms and constrained hydrogen atom parameters converged smoothly to *R* = 0.0315 for *F* ^2^>2σ, but a difference Fourier synthesis at this stage showed unacceptable features, with a hole of −1.0 e/A^3^ and two peaks of 0.7 e/A^3^ near Cu2, while there were no significant peaks or holes near Cu1. In addition, the temperature factors for Cu2 indicated an ellipsoid much elongated compared to that for Cu1 (Fig. 3 ▶). In case these features were related to systematic anisotropies that might have existed in the data collection, a trial was made to apply a smoothly varying scale factor by a 12 parameter model with *XABS2* (Parkin *et al.*, 1995 ▶). This had no significant effect on either the difference Fourier map or the *R* values, and the trial was abandoned. Instead, a model with Cu2 disordered equally between two positions was refined, which converged at *R* = 0.0307 for *F* ^2^>2σ, and showed maximum and minimum residual electron densities at 0.71 and −0.81 e/A^3^ near Cu2*B* and Cu2*A*, respectively, indicating that the sites were not equally occupied. Allowing the occupancy factors to vary led to the final model, with *R* = 0.0265 for *F* ^2^>2σ, and residual electron density maxima of 0.29 and −0.31 e/A^3^ near S and P atoms. The disordered Cu^II^ atoms sites are 0.524 (4) Å apart, with occupancy factors of 64.7 (6)% and 35.3 (6)%. To facilitate convergence, the *U*_ij_ for the disordered Cu atoms were constrained to be identical. It is likely that S2 could also be disordered, reflecting bonding to the two different Cu2 sites. We have not pursued attempts to model this.

The two partial copper positions might have represented alternating sites in a larger unit cell with the short *a* axis doubled. This would have made the disorder an artifact due to the data collection in that only reflections with *h* = 2*n* would have been collected. However, inspection of precession photographs of the *h*0*l*, *h*1*l* and *h*2*l* layers did not reveal any indication of doubling of the *a* axis. Furthermore, if that had been the case, the occupancies of the disorder components would have refined to approximately 0.5 rather than 0.647 (6) and 0.353 (6).

H atoms were constrained to idealized positions with C—H distances of 0.96 Å. The orientations of the methyl groups were determined by calculation of electron density in the toroid that should contain the H atoms of the idealized methyl groups. The *U*_eq_ values for the H atoms were fixed at 1.2 times the *U*_iso_ of their bonded C atoms.

Values for the Goodness of Fit (GOOF) near the end of the refinements were rather low, at 0.66, implying that at least some of the estimated σ values for the data were too high. The factor *p* in the data processing (Corfield *et al.*, 1973 ▶) had originally been set at 0.06, a value that now seemed too large for such a highly refined structure. The σ values were adjusted to correspond to p = 0.05 with the equation: [σ(new)/*F* ^2^]^2^ = [σ(old)/*F* ^2^]^2^−(0.06^2^−0.05^2^). In addition, σ values for 182 very weak reflections, which had been grossly overestimated previously, were set equal to the average value found for the 145 reflections observed with *I*<0. (These reflections were set to *F* ^2^ = 0.) Final refinements with these adjustments to the σ values raised the value of the GOOF to 0.79 with no significant changes to any parameters.

Crystal data, data collection and structure refinement details are summarized in Table 2 ▶.

## Supplementary Material

Crystal structure: contains datablock(s) I. DOI: 10.1107/S1600536814021412/pk2530sup1.cif

Structure factors: contains datablock(s) I. DOI: 10.1107/S1600536814021412/pk2530Isup2.hkl

CCDC reference: 1026492

Additional supporting information:  crystallographic information; 3D view; checkCIF report

## Figures and Tables

**Figure 2:**
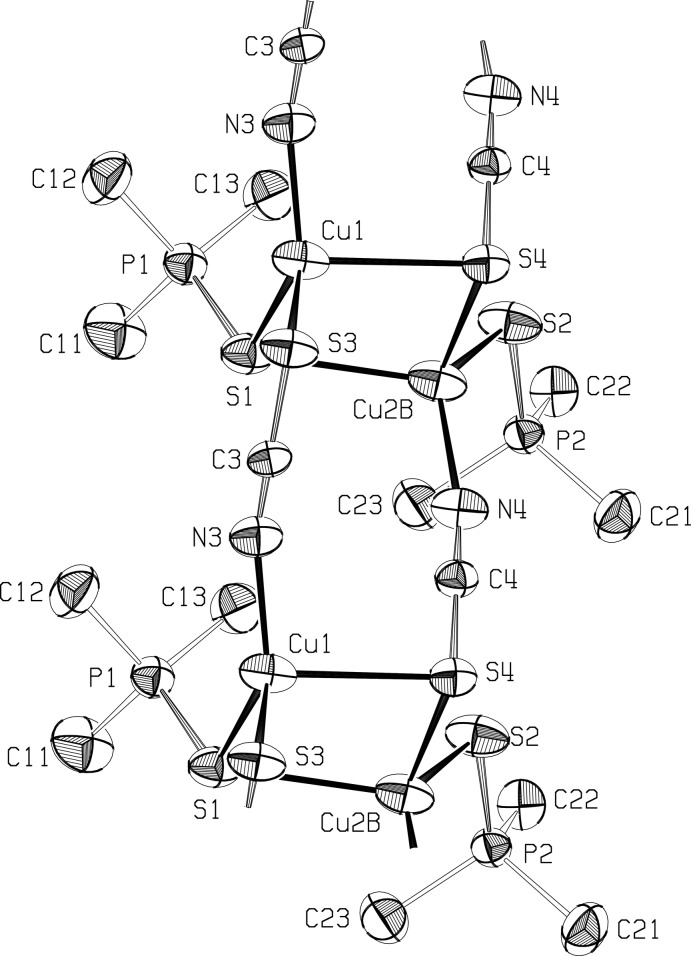
The alternate ribbon structure of the title polymer, showing the environment of Cu2 in position *B*, with ellipsoids at the 50% level. Hydrogen atoms omitted.

**Table 1:** Selected geometric parameters (, )

Cu1N3	1.943(3)	Cu2*B*N4^i^	1.969(6)
Cu1S1	2.2830(11)	Cu2*B*S2	2.315(5)
Cu1S3	2.3431(11)	Cu2*B*S4	2.416(5)
Cu2*A*N4^i^	1.894(4)	S1P1	1.9935(13)
Cu2*A*S2	2.206(3)	S2P2	1.9848(14)
Cu2*A*S4	2.316(3)		
			
P1S1Cu1	105.99(5)	C4S4Cu2*A*	102.18(13)
P2S2Cu2*A*	106.09(8)	C3N3Cu1	164.6(3)
P2S2Cu2*B*	106.55(13)	C4N4Cu2*A* ^ii^	169.9(3)
C3^i^S3Cu1	102.90(12)	C4N4Cu2*B* ^ii^	166.1(3)
C4S4Cu2*B*	100.44(16)		

Symmetry codes: (i) 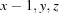; (ii) 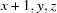.

**Table 2:** Experimental details

Crystal data	
Chemical formula	[Cu(NCS)(C_3_H_9_PS)]
*M*_r_	229.75
Crystal system, space group	Monoclinic, *P*2_1_/*c*
Temperature (K)	298
*a*, *b*, *c* ()	5.793(3), 14.091(3), 22.064(7)
()	98.945(17)
*V* (^3^)	1779.2(11)
*Z*	8
Radiation type	Cu *K*
(mm^1^)	8.73
Crystal size (mm)	0.31 0.06 0.05

Data collection	
Diffractometer	Picker 4-circle
Absorption correction	Gaussian (Busing Levy, 1957 ▶)
*T*_min_, *T*_max_	0.433, 0.704
No. of measured, independent and observed [*I* > 2(*I*)] reflections	6315, 2912, 2144
*R*_int_	0.058
(sin /)_max_ (^1^)	0.579

Refinement	
*R*[*F* ^2^ > 2(*F* ^2^)], *wR*(*F* ^2^), *S*	0.027, 0.076, 0.79
No. of reflections	2912
No. of parameters	174
H-atom treatment	H-atom parameters constrained
_max_, _min_ (e ^3^)	0.29, 0.31

Computer programs: Corfield (1972 ▶), *SHELXL97* (Sheldrick, 2008 ▶) and *ORTEPIII* (Burnett Johnson, 1996 ▶). Data reduction followed procedures in Corfield *et al.* (1973 ▶) with *p* = 0.06, with programs written by Corfield and by Graeme Gainsford, local superposition program (Corfield, 1972 ▶).

## supplementary crystallographic information

### Crystal data

**Table:**

[Cu(NCS)(C_3_H_9_PS)]	*F*(000) = 928
*M**_r_* = 229.75	*D*_x_ = 1.715 Mg m^−^^3^*D*_m_ = 1.709 Mg m^−^^3^*D*_m_ measured by flotation
Monoclinic, *P*2_1_/*c*	Cu *K*α radiation, λ = 1.5418 Å
Hall symbol: -P 2ybc	Cell parameters from 19 reflections
*a* = 5.793 (3) Å	θ = 6.4–41.0°
*b* = 14.091 (3) Å	µ = 8.73 mm^−^^1^
*c* = 22.064 (7) Å	*T* = 298 K
β = 98.945 (17)°	Rod, colorless
*V* = 1779.2 (11) Å^3^	0.31 × 0.06 × 0.05 mm
*Z* = 8	

### Data collection

**Table:**

Picker 4-circle diffractometer	2144 reflections with *I* > 2σ(*I*)
Radiation source: sealed X-ray tube	*R*_int_ = 0.058
Oriented graphite 200 reflection monochromator	θ_max_ = 63.3°, θ_min_ = 3.7°
θ/2θ scans	*h* = 0→6
Absorption correction: gaussian (Busing & Levy, 1957)	*k* = −16→16
*T*_min_ = 0.433, *T*_max_ = 0.704	*l* = −25→25
6315 measured reflections	6 standard reflections every 400 reflections
2912 independent reflections	intensity decay: 0.6(4)

### Refinement

**Table:**

Refinement on *F*^2^	Secondary atom site location: difference Fourier map
Least-squares matrix: full	Hydrogen site location: inferred from neighbouring sites
*R*[*F*^2^ > 2σ(*F*^2^)] = 0.027	H-atom parameters constrained
*wR*(*F*^2^) = 0.076	*w* = 1/[σ^2^(*F*_o_^2^)]
*S* = 0.79	(Δ/σ)_max_ = 0.001
2912 reflections	Δρ_max_ = 0.29 e Å^−^^3^
174 parameters	Δρ_min_ = −0.31 e Å^−^^3^
0 restraints	Extinction correction: *SHELXL97* (Sheldrick, 2008), Fc^*^=kFc[1+0.001xFc^2^λ^3^/sin(2θ)]^-1/4^
Primary atom site location: structure-invariant direct methods	Extinction coefficient: 0.00128 (9)

### Special details

**Table:**

Geometry. All e.s.d.'s (except the e.s.d. in the dihedral angle between two l.s. planes) are estimated using the full covariance matrix. The cell e.s.d.'s are taken into account individually in the estimation of e.s.d.'s in distances, angles and torsion angles; correlations between e.s.d.'s in cell parameters are only used when they are defined by crystal symmetry. An approximate (isotropic) treatment of cell e.s.d.'s is used for estimating e.s.d.'s involving l.s. planes.
Refinement. Refinement of *F*^2^ against ALL reflections. The weighted *R*-factor *wR* and goodness of fit *S* are based on *F*^2^, conventional *R*-factors *R* are based on *F*, with *F* set to zero for negative *F*^2^. The threshold expression of *F*^2^ > σ(*F*^2^) is used only for calculating *R*-factors(gt) *etc*. and is not relevant to the choice of reflections for refinement. *R*-factors based on *F*^2^ are statistically about twice as large as those based on *F*, and *R*-factors based on ALL data will be even larger.

### Fractional atomic coordinates and isotropic or equivalent isotropic displacement parameters (Å^2^)

**Table:**

	*x*	*y*	*z*	*U*_iso_*/*U*_eq_	Occ. (<1)
Cu1	0.03939 (9)	0.30233 (4)	0.41293 (3)	0.04640 (17)	
Cu2A	−0.2710 (4)	0.3110 (2)	0.25554 (15)	0.0484 (5)	0.647 (6)
Cu2B	−0.2599 (8)	0.3379 (3)	0.2721 (2)	0.0484 (5)	0.353 (6)
S1	−0.17677 (15)	0.43301 (6)	0.37964 (4)	0.0432 (2)	
S2	−0.11907 (16)	0.39160 (7)	0.18567 (4)	0.0524 (3)	
P1	0.05098 (15)	0.53894 (6)	0.37911 (4)	0.0356 (2)	
P2	−0.38568 (14)	0.45343 (6)	0.13294 (4)	0.0326 (2)	
S3	−0.18043 (14)	0.20640 (6)	0.46933 (4)	0.0440 (2)	
S4	0.01342 (14)	0.21297 (6)	0.30788 (4)	0.0372 (2)	
N3	0.3696 (5)	0.2829 (2)	0.44265 (13)	0.0448 (7)	
C3	0.5556 (5)	0.2523 (2)	0.45340 (14)	0.0326 (7)	
N4	0.4265 (5)	0.2852 (2)	0.27391 (14)	0.0488 (8)	
C4	0.2553 (5)	0.2565 (2)	0.28759 (14)	0.0341 (7)	
C11	−0.1011 (7)	0.6494 (3)	0.3727 (2)	0.0656 (12)	
H11A	0.0079	0.7001	0.3705	0.098*	
H11B	−0.1773	0.6582	0.4080	0.098*	
H11C	−0.2157	0.6492	0.3363	0.098*	
C12	0.2638 (7)	0.5438 (3)	0.44721 (16)	0.0566 (11)	
H12A	0.3472	0.4847	0.4523	0.085*	
H12B	0.1868	0.5547	0.4821	0.085*	
H12C	0.3716	0.5945	0.4438	0.085*	
C13	0.2128 (7)	0.5328 (3)	0.31678 (16)	0.0555 (10)	
H13A	0.1069	0.5336	0.2787	0.083*	
H13B	0.3024	0.4752	0.3197	0.083*	
H13C	0.3162	0.5863	0.3184	0.083*	
C21	−0.5941 (7)	0.3702 (3)	0.09550 (17)	0.0572 (11)	
H21A	−0.7119	0.4035	0.0681	0.086*	
H21B	−0.6657	0.3369	0.1257	0.086*	
H21C	−0.5165	0.3257	0.0726	0.086*	
C22	−0.2800 (6)	0.5203 (2)	0.07406 (15)	0.0443 (8)	
H22A	−0.4071	0.5546	0.0507	0.066*	
H22B	−0.2131	0.4780	0.0474	0.066*	
H22C	−0.1630	0.5643	0.0924	0.066*	
C23	−0.5452 (7)	0.5329 (3)	0.17392 (18)	0.0563 (10)	
H23A	−0.4407	0.5795	0.1947	0.084*	
H23B	−0.6165	0.4979	0.2034	0.084*	
H23C	−0.6642	0.5639	0.1457	0.084*	

### Atomic displacement parameters (Å^2^)

**Table:**

	*U*^11^	*U*^22^	*U*^33^	*U*^12^	*U*^13^	*U*^23^
Cu1	0.0311 (3)	0.0459 (3)	0.0631 (4)	0.0036 (2)	0.0102 (2)	0.0118 (3)
Cu2A	0.0351 (4)	0.0514 (13)	0.0613 (13)	0.0032 (7)	0.0154 (7)	0.0154 (8)
Cu2B	0.0351 (4)	0.0514 (13)	0.0613 (13)	0.0032 (7)	0.0154 (7)	0.0154 (8)
S1	0.0335 (5)	0.0360 (5)	0.0598 (6)	0.0020 (4)	0.0066 (4)	0.0080 (4)
S2	0.0374 (5)	0.0667 (7)	0.0550 (6)	0.0076 (4)	0.0128 (4)	0.0239 (5)
P1	0.0355 (5)	0.0327 (4)	0.0385 (5)	0.0027 (4)	0.0053 (4)	0.0023 (4)
P2	0.0317 (4)	0.0317 (4)	0.0356 (4)	−0.0027 (3)	0.0088 (3)	−0.0013 (3)
S3	0.0317 (5)	0.0437 (5)	0.0581 (5)	0.0036 (4)	0.0117 (4)	0.0168 (4)
S4	0.0306 (4)	0.0380 (4)	0.0429 (5)	−0.0009 (3)	0.0058 (3)	0.0048 (4)
N3	0.0340 (17)	0.0473 (18)	0.0534 (18)	−0.0045 (14)	0.0075 (14)	0.0049 (14)
C3	0.0264 (17)	0.0344 (17)	0.0371 (17)	−0.0046 (14)	0.0053 (13)	0.0029 (14)
N4	0.0347 (17)	0.0549 (19)	0.0571 (19)	0.0040 (14)	0.0083 (14)	0.0100 (15)
C4	0.0294 (17)	0.0348 (18)	0.0373 (18)	0.0035 (14)	0.0031 (14)	0.0017 (14)
C11	0.066 (3)	0.035 (2)	0.097 (3)	0.0106 (19)	0.015 (2)	0.009 (2)
C12	0.058 (3)	0.068 (3)	0.041 (2)	−0.007 (2)	−0.0039 (18)	−0.0054 (19)
C13	0.052 (2)	0.072 (3)	0.043 (2)	−0.005 (2)	0.0114 (17)	0.0016 (19)
C21	0.056 (2)	0.060 (3)	0.056 (2)	−0.024 (2)	0.0123 (19)	−0.015 (2)
C22	0.051 (2)	0.041 (2)	0.0412 (19)	−0.0013 (17)	0.0092 (16)	0.0050 (16)
C23	0.055 (2)	0.052 (2)	0.065 (3)	0.0013 (19)	0.020 (2)	−0.011 (2)

### Geometric parameters (Å, º)

**Table:**

Cu1—N3	1.943 (3)	S4—C4	1.654 (3)
Cu1—S1	2.2830 (11)	N3—C3	1.150 (4)
Cu1—S3	2.3431 (11)	N4—C4	1.154 (4)
Cu1—S4	2.6214 (12)	C11—H11A	0.9600
Cu1—Cu2B	3.351 (5)	C11—H11B	0.9600
Cu1—Cu2A	3.656 (3)	C11—H11C	0.9600
Cu2A—N4^i^	1.894 (4)	C12—H12A	0.9600
Cu2A—S2	2.206 (3)	C12—H12B	0.9600
Cu2A—S4	2.316 (3)	C12—H12C	0.9600
Cu2B—N4^i^	1.969 (6)	C13—H13A	0.9600
Cu2B—S2	2.315 (5)	C13—H13B	0.9600
Cu2B—S4	2.416 (5)	C13—H13C	0.9600
Cu2B—S1	2.702 (5)	C21—H21A	0.9600
S1—P1	1.9935 (13)	C21—H21B	0.9600
S2—P2	1.9848 (14)	C21—H21C	0.9600
P1—C13	1.783 (4)	C22—H22A	0.9600
P1—C11	1.783 (4)	C22—H22B	0.9600
P1—C12	1.791 (3)	C22—H22C	0.9600
P2—C23	1.784 (4)	C23—H23A	0.9600
P2—C22	1.788 (3)	C23—H23B	0.9600
P2—C21	1.790 (3)	C23—H23C	0.9600
S3—C3^i^	1.648 (3)		
			
N3—Cu1—S1	133.19 (9)	C4—N4—Cu2A^ii^	169.9 (3)
N3—Cu1—S3	109.10 (9)	C4—N4—Cu2B^ii^	166.1 (3)
S1—Cu1—S3	108.66 (5)	Cu2A^ii^—N4—Cu2B^ii^	15.43 (11)
N3—Cu1—S4	98.56 (9)	N4—C4—S4	178.6 (3)
S1—Cu1—S4	98.61 (4)	P1—C11—H11A	109.5
S3—Cu1—S4	103.37 (4)	P1—C11—H11B	109.5
N4^i^—Cu2A—S2	137.01 (15)	H11A—C11—H11B	109.5
N4^i^—Cu2A—S4	112.91 (14)	P1—C11—H11C	109.5
S2—Cu2A—S4	108.98 (10)	H11A—C11—H11C	109.5
N4^i^—Cu2B—S2	125.7 (3)	H11B—C11—H11C	109.5
N4^i^—Cu2B—S4	106.2 (2)	P1—C12—H12A	109.5
S2—Cu2B—S4	102.14 (18)	P1—C12—H12B	109.5
N4^i^—Cu2B—S1	102.0 (2)	H12A—C12—H12B	109.5
S2—Cu2B—S1	121.57 (19)	P1—C12—H12C	109.5
S4—Cu2B—S1	93.24 (16)	H12A—C12—H12C	109.5
P1—S1—Cu1	105.99 (5)	H12B—C12—H12C	109.5
P1—S1—Cu2B	113.21 (11)	P1—C13—H13A	109.5
Cu1—S1—Cu2B	84.04 (11)	P1—C13—H13B	109.5
P2—S2—Cu2A	106.09 (8)	H13A—C13—H13B	109.5
P2—S2—Cu2B	106.55 (13)	P1—C13—H13C	109.5
C13—P1—C11	107.1 (2)	H13A—C13—H13C	109.5
C13—P1—C12	105.85 (18)	H13B—C13—H13C	109.5
C11—P1—C12	107.5 (2)	P2—C21—H21A	109.5
C13—P1—S1	113.33 (14)	P2—C21—H21B	109.5
C11—P1—S1	109.55 (15)	H21A—C21—H21B	109.5
C12—P1—S1	113.18 (14)	P2—C21—H21C	109.5
C23—P2—C22	107.47 (18)	H21A—C21—H21C	109.5
C23—P2—C21	106.22 (19)	H21B—C21—H21C	109.5
C22—P2—C21	107.02 (17)	P2—C22—H22A	109.5
C23—P2—S2	113.29 (14)	P2—C22—H22B	109.5
C22—P2—S2	109.52 (12)	H22A—C22—H22B	109.5
C21—P2—S2	112.98 (15)	P2—C22—H22C	109.5
C3^i^—S3—Cu1	102.90 (12)	H22A—C22—H22C	109.5
C4—S4—Cu2B	100.44 (16)	H22B—C22—H22C	109.5
C4—S4—Cu2A	102.18 (13)	P2—C23—H23A	109.5
C4—S4—Cu1	97.27 (12)	P2—C23—H23B	109.5
Cu2B—S4—Cu1	83.30 (12)	H23A—C23—H23B	109.5
Cu2A—S4—Cu1	95.33 (10)	P2—C23—H23C	109.5
C3—N3—Cu1	164.6 (3)	H23A—C23—H23C	109.5
N3—C3—S3^ii^	178.8 (3)	H23B—C23—H23C	109.5

Symmetry codes: (i) *x*−1, *y*, *z*; (ii) *x*+1, *y*, *z*.

## References

[bb1] Burnett, M. N. & Johnson, C. K. (1996). *ORTEPIII*. Report ORNL-6895. Oak Ridge National Laboratory, Tennessee, USA.

[bb2] Busing, W. R. & Levy, H. A. (1957). *Acta Cryst.* **10**, 180–182.

[bb3] Corfield, P. W. R. (1972). Local versions of standard programs, written at Ohio State University.

[bb4] Corfield, P. W. R., Dabrowiak, J. C. & Gore, E. S. (1973). *Inorg. Chem.* **12**, 1734–1740.

[bb5] Eller, P. G. & Corfield, P. W. R. (1971). *J. Chem. Soc. Chem. Commun.* pp. 105–106.

[bb6] Healy, P. C., Pakawatchai, C., Papasergio, R. I., Patrick, V. A. & White, A. H. (1984). *Inorg. Chem.* **23**, 3769–3776.

[bb7] Machura, B., Świtlicka, A., Mroziński, J., Kalińska, B. & Kruszynski, R. (2013). *Polyhedron*, **52**, 1276–1286.

[bb8] Miller, K. M., McCullough, S. M., Lepekhina, E. A., Thibau, I. J., Pike, R. D., Li, X., Killarney, J. P. & Patterson, H. H. (2011). *Inorg. Chem.* **50**, 7239–7249. 10.1021/ic200821f 21728324

[bb9] Niu, Y.-Y., Wu, B.-L., Guo, X.-L., Song, Y.-L., Liu, X.-C., Zhang, H.-Y., Hou, H.-W., Niu, C.-Y. & Ng, S.-W. (2008). *Cryst. Growth Des.* **8**, 2393–2401.

[bb10] Parkin, S., Moezzi, B. & Hope, H. (1995). *J. Appl. Cryst.* **28**, 53–56.

[bb11] Raston, C. L., Walter, B. & White, A. H. (1979). *Aust. J. Chem.* **32**, 2757–2761.

[bb12] Sheldrick, G. M. (2008). *Acta Cryst.* A**64**, 112–122. 10.1107/S0108767307043930 18156677

[bb13] Sues, E. S., Lough, A. J. & Morris, R. H. (2014). *Chem. Commun.* **50**, 4707–4710. 10.1039/c3cc49456j 24675688

[bb14] Tiedemann, M. A., Mandell, C. L., Chan, B. C. & Nataro, C. (2014). *Inorg. Chim. Acta*. 10.1016/j. ica. 2014.06.004.

[bb15] Tiethof, J. A., Hetey, A. T. & Meek, D. W. (1974). *Inorg. Chem.* **13**, 2505–2509.

[bb16] Tiethof, J. A., Stalick, J. K. & Meek, D. W. (1973). *Inorg. Chem.* **12**, 1170–1174.

